# Maternal Health Status Correlates with Nest Success of Leatherback Sea Turtles (*Dermochelys coriacea*) from Florida

**DOI:** 10.1371/journal.pone.0031841

**Published:** 2012-02-16

**Authors:** Justin R. Perrault, Debra L. Miller, Erica Eads, Chris Johnson, Anita Merrill, Larry J. Thompson, Jeanette Wyneken

**Affiliations:** 1 Department of Biological Sciences, Florida Atlantic University, Boca Raton, Florida, United States of America; 2 The University of Georgia, College of Veterinary Medicine, Veterinary Diagnostic and Investigational Laboratory, Tifton, Georgia, United States of America; 3 College of Veterinary Medicine, University of Tennessee, Knoxville, Tennessee, United States of America; 4 Loggerhead Marinelife Center, Juno Beach, Florida, United States of America; 5 Nestlé Purina PetCare, St. Louis, Missouri, United States of America; Monash University, Australia

## Abstract

Of the seven sea turtle species, the critically endangered leatherback sea turtle (*Dermochelys coriacea*) exhibits the lowest and most variable nest success (i.e., hatching success and emergence success) for reasons that remain largely unknown. In an attempt to identify or rule out causes of low reproductive success in this species, we established the largest sample size (n = 60–70 for most values) of baseline blood parameters (protein electrophoresis, hematology, plasma biochemistry) for this species to date. Hematologic, protein electrophoretic and biochemical values are important tools that can provide information regarding the physiological condition of an individual and population health as a whole. It has been proposed that the health of nesting individuals affects their reproductive output. In order to establish correlations with low reproductive success in leatherback sea turtles from Florida, we compared maternal health indices to hatching success and emergence success of their nests. As expected, hatching success (median = 57.4%) and emergence success (median = 49.1%) in Floridian leatherbacks were low during the study period (2007–2008 nesting seasons), a trend common in most nesting leatherback populations (average global hatching success = ∼50%). One protein electrophoretic value (gamma globulin protein) and one hematologic value (red blood cell count) significantly correlated with hatching success and emergence success. Several maternal biochemical parameters correlated with hatching success and/or emergence success including alkaline phosphatase activity, blood urea nitrogen, calcium, calcium∶phosphorus ratio, carbon dioxide, cholesterol, creatinine, and phosphorus. Our results suggest that in leatherbacks, physiological parameters correlate with hatching success and emergence success of their nests. We conclude that long-term and comparative studies are needed to determine if certain individuals produce nests with lower hatching success and emergence success than others, and if those individuals with evidence of chronic suboptimal health have lower reproductive success.

## Introduction

Few studies broadly report baseline blood values (i.e., hematology, plasma biochemistry, plasma protein electrophoresis) in the critically endangered leatherback sea turtle (*Dermochelys coriacea*) [1]–[5]. Health assessments and long-term health monitoring studies are critical for endangered species [6]. Normal or expected ranges of blood values for wildlife are lacking in the literature and these data are vital for population comparisons and management techniques and actions, as well as assessment of the health of an individual animal [6]. Health indices are useful for wildlife species in that they provide information regarding the physiological or nutritional status of an individual, as well as aiding in diagnosis of disease(s) [7]. These baseline hematologic measures of wildlife health provide a window into overall health.

Establishing and understanding both health status and health-related problems allow for more sound conservation and management approaches. Existing ranges of biochemical values for reptiles include those of a variety of turtles, tortoises, sea turtles, snakes, and lizards [8], [9]. Blood profiles for “clinically normal” (normal behavior and apparent physical condition) Florida green (*Chelonia mydas*) and loggerhead (*Caretta caretta*) sea turtles are available [10]. Blood collection is common in sea turtle studies and is often used in population genetics to identify management units [11]. Published blood parameters are limited in sample size and population/geographic coverage for leatherback turtles: nesting females from Gabon, Africa [1], Trinidad [2], Papua New Guinea [4], Costa Rica [4], St. Croix, USVI [4], Equatorial Guinea [5], wild-caught and entangled males and females from Georgia & Massachusetts, USA [3], and Pacific foraging males and females [4].

The leatherback turtle is listed as critically endangered internationally by the IUCN [12]. Of the seven sea turtle species, the leatherback has the lowest average global nest success (∼50%), which we define here as hatching success and hatchling emergence success; their nests also exhibit high early embryonic mortality in some populations [13]–[16]. Lower hatching success and emergence success in the leatherback turtle has yet to be explained; however, Bell et al. [13] hypothesized that maternal reproductive health, chemical contaminants, or bacterial infection may contribute to low reproductive success. In hatchling leatherbacks from Florida, both a necessary nutrient (selenium) and a chemical contaminant (mercury) correlated with hatching success and emergence success [17]. In other sea turtle species, physical factors such as nest higher temperatures [18], larger sand grain sizes [19], and tidal inundation [20] can reduce sea turtle hatching success and increase in-nest mortality.

Leatherbacks lay 6–11 clutches per season (at 8–11 day intervals) with an average of 70–80 eggs deposited in each nest [13], [14], [21]. When a marine turtle arrives at the nesting beach, the ovaries are mature and all preovulatory follicles that will be ovulated during that nesting season are mature [22], [23]. The albumen and eggshell, however, are formed progressively for each clutch (shell does not completely form until 7 or more days post-ovulation) during the nesting season [21]. Individuals vary in their nesting beach philopatry so that some turtles use the same beach for all their nests while others scatter their nests among several beaches along the Florida coast [14]. Each clutch is deposited in a nest dug in the sand by the female, then the eggs are covered and the nest is abandoned to incubate and hatch unattended. Hatchlings emerge several days after hatching by digging out of the nest, often as a group.

Because there is no post-hatchling parental care in sea turtles, the embryos must survive by relying on the nutritional energy reserves (i.e., yolk and albumen) provided by the mother. An individual with suboptimal health may produce inadequately provisioned eggs with decreased nutritional reserves, which may affect the survival of the offspring. In an attempt to understand low reproductive success of the leatherback turtle, our study examined baseline blood parameters (as indicators of health) for an assemblage of nesting female leatherbacks in Palm Beach County, Florida, USA. These parameters were then correlated to hatching success and emergence success of their clutches to determine if maternal health status influenced either measure of nest success. Standard blood parameters (plasma protein electrophoresis, hematology, plasma biochemistry) were used to evaluate the health status of this nesting population.

## Materials and Methods

### Ethics Statement

Our study was carried out in accordance with Florida Fish and Wildlife Conservation Commission Marine Turtle Permit #073. Florida Atlantic University's Institutional Animal Care and Use Committee (IACUC) approved this study (protocol #A07-03).

### 1. Study Period and Site Description

**Figure 1:**
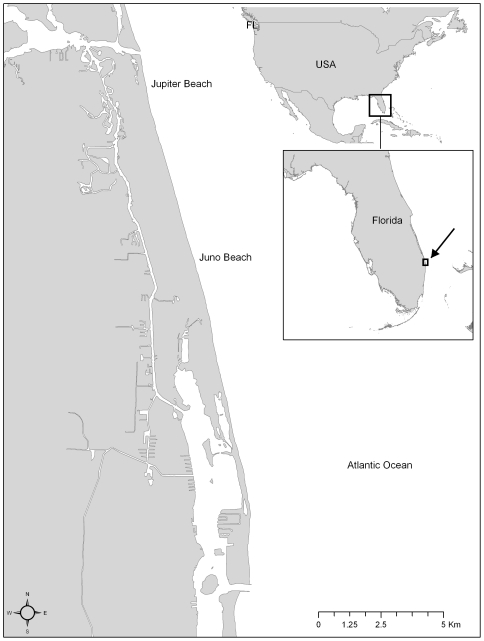
Map of the nesting beach extending across Juno Beach and Jupiter Beach, Florida, USA.

Nesting leatherback sea turtles were sampled during the 2007 and 2008 nesting seasons on the 20 km nesting beach. This semi-urban area is located between the Jupiter Inlet (26°56′36″N, 80°04′15″W) and the Lake Worth Inlet (26°46′24″N, 80°01′53″W, **Fig. 1**). Nesting commences in late February-early March, peaks in May, and usually ends in late June-early July [14]. The Loggerhead Marinelife Center (LMC, Juno Beach, FL, USA) has performed nightly monitoring of leatherback activity on this nesting beach since 2001 [24]. Approximately 460 leatherbacks have been tagged at this rookery since tagging began [K. Martin, pers. comm.].

### 2. Sample collection from nesting turtles

The nesting beach was monitored nightly (a collaborative effort with the LMC's saturation tagging project) on foot or with all-terrain vehicles to encounter leatherbacks during their nesting season. Individual nesting females were identified by flipper tags and/or passive integrated transponder (PIT) tags. Nesting females were approached after egg deposition commenced, once they entered the nesting fixed action pattern during which they are nonresponsive to manipulation [25]. The venipuncture site was cleaned with a 70% isopropyl alcohol swab before insertion of the pre-coated heparinized needle. Approximately 7 mLs of blood were collected from the femoral rete system [11], [26] aseptically using a 20 G 1½ Monoject® (Sherwood Medical, St. Louis, MO, USA) venous collection needle fitted in a BD vacutainer® tube holder. Blood was collected in BD plasma-separator and lithium-heparin (LH) vacutainer® tubes (Becton, Dickinson and Co., Franklin Lakes, NJ, USA) or using a BD Precisionglide® needle (21 G, 2 inch), pre-coated with sodium-heparin (Elkins-Sinn, Inc., Cherry Hill, NJ, USA) and a 10 mL BD Luer-Lok™ syringe to fill blood vacutainers® and prepare 2 blood-smear slides. After blood collection, the venipuncture site was disinfected with a new alcohol swab and pressure was applied to minimize further bleeding. Blood was kept on ice immediately after collection. Plasma-separator tubes were spun in the field using a centrifuge at 3500 rpms for 5 minutes, approximately 1 hour after blood collection. Samples were collected from the same nesting female during her subsequent nesting events (>8–11 days later) when possible. Plasma was frozen at −20°C and blood in the LH tubes was refrigerated for 12–36 h until they were shipped overnight, on ice, to the Veterinary Diagnostic and Investigational Laboratory at the University of Georgia (UGA-VDIL, Tifton, GA, USA) for analyses.

After sample collection, the turtle's size and body condition were visually assessed. The presence of injuries, scars and epibionts were noted. The turtles' minimum curved carapace length was recorded (from nuchal notch to posterior tip of the caudal peduncle, CCL_min_) [27].

### 3. Nest Inventory

Eggs hatch and hatchlings emerge from nests between June-late August. Three days following the mass emergence event, nests from the study females were excavated and inventoried to determine hatching success and emergence success [28]:

HATCHING SUCCESS:
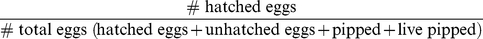
EMERGENCE SUCCESS:
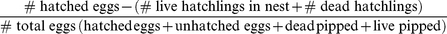
Only yolked eggs were included in calculations of hatching success and emergence success. Any depredated, inundated, or washed out nests (due to storm) were noted and not included in statistical analyses.

### 4. Sample processing

Plasma samples were used for biochemical analyses, which were carried out at the UGA-VDIL using a Bayer Advia® 1200 Chemistry System (*S*iemens Medical Solutions Diagnostics, Tarrytown, NY, USA). Plasma was analyzed for alanine aminotransferase activity (ALT), alkaline phosphatase activity (ALKP), amylase activity, anion gap, aspartate aminotransferase activity (AST), bile acids, blood urea nitrogen (BUN), calcium, calcium∶phosphorus (Ca∶P) ratio, carbon dioxide (CO_2_), chloride, cholesterol, creatine kinase activity (CK), creatinine, glucose, iron, lactate dehydrogenase activity (LDH), lipase activity, phosphorus, potassium, sodium, thyroxine, total protein, and uric acid.

Packed cell volume (PCV) was calculated from a subsample of blood that was collected from the LH tube into a microhematocrit capillary tube (Fisher HealthCare, Houston, TX, USA) and Critoseal® (Sherwood Medical Co., Deland, FL, USA) was used as the sealant. A Microspin 24 micro-centrifuge (Vulcan Technologies, Orlando, FL, USA) was used to spin the hematocrit tube and a Damon/IEC Division microcapillary hematocrit reader (Damon/IEC Division, Needham, MA, USA) was used to measure PCV. Blood cell counts (RBC and WBC) and thrombocyte counts were determined using the Natt-Herrick Cell Count Method [29]. Blood smears were fixed in methanol and stained using the Wright-Giemsa procedure at the UGA-VDIL for differential leukocyte counts. Differential leukocytes counts were based on examination of 100 total leukocytes.

Total protein was quantified from plasma by electrophoresis using the Helena SPIFE® 3000 system (Split Beta SPE, Helena Laboratories, Beaumont, TX, USA). The fractions determined include pre-albumin, albumin, and the globulin proteins (total alpha, beta, and gamma).

### 5. Statistical Analyses

All statistical analyses were performed using SYSTAT v. 12.2 (Systat, Inc., Evanston, IL). The Shapiro-Wilk statistic was used to determine if the data were normally distributed. Mean ± standard deviation are reported for normally distributed data. For data not meeting normality assumptions, the median is reported. Ranges are reported for all data. For nesting females sampled more than once, repeated measures ANOVAs were used to determine if the measured blood parameters differed significantly from the initial to subsequent nesting encounter. Linear least-squares regressions were used to determine if any health parameters decreased as the season progressed (independent variable = date of nesting encounter). To correlate hatching success and emergence success with measured maternal baseline blood parameters, least-squares linear regressions were used for normally distributed data. Rank regressions were used for data that could not meet normality assumptions [30]. For some biochemical parameters, the nests with 0% success were removed from the regression analyses, as most likely individuals in those nests died due to environmental factors (i.e., water table, inundation, etc.).

## Results

### 1. Physical Examinations

A total of 60 individual nesting female leatherback turtles (40 in 2007 and 20 in 2008) were sampled throughout the project. Ten nesting females were sampled twice (6 in 2007, 4 in 2008) and 1 individual was sampled 3 times (2007). Average minimum curved carapace length (CCL_min_) was 153.1±7.6 cm with a range of 135–174 cm. Acorn barnacles (*Balanus* sp.) were present on all sampled turtles. One individual had a large (∼20 cm), open and bleeding laceration on the left shoulder, while 30 turtles had smaller (∼2–13 cm), fresh injuries on the flippers and/or carapace (consistent with propeller wounds, shark attack lacerations, courtship wounds, or evidence of fisheries interactions, such as hooks and/or ligature scars). One of the 60 turtles was missing its right rear flipper (past trauma, healed), two turtles had partial amputations (partially healed) of their left rear flippers (posterior margin and distal phalanges), one turtle had a partial amputation of its right rear flipper (partially healed), one turtle had a partial amputation (distal, partially healed) of its right front flipper, and another turtle was missing ∼30% of both right and left front flippers (trailing edges, undergoing repair). Nearly all sampled individuals had scars or small wounds (undergoing repair) on their carapace and/or appendages.

### 2. Nest Fate

During nest inventory, 13 nests were excluded from the study (3 washed out, 2 were depredated, 8 could not be located during excavations) and were not included in analyses. The number of yolked eggs laid by each nesting female ranged from 34–103 (n = 72, 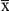
±SD = 74±14 eggs). Hatching success ranged from 0–93.4% (n = 72, median = 57.4%). Emergence success ranged from 0–93.4% (n = 72, median = 49.1%). Hatching success (rank regression, n = 72, r^2^ = 0.14, p = 0.001) and emergence success (rank regression, n = 72, r^2^ = 0.14, p = 0.001) both negatively correlated with date of nesting.

### 3. Health Parameters

The results of electrophoretic tests are presented in **Table 1**. Protein electrophoretic results from other locations are included for comparison. **Table 2** summarizes the hematologic results (other locations are included for comparison, **Table S1**) and **Table 3** summarizes the plasma biochemical test results (other locations are included for comparison, **Tables S2, S3 & S4**). The only biochemical value that significantly changed (decreased) from the initial nesting encounter to the next encounter was calcium (average decrease of 0.39 mg/dL, F_1,10_ = 5.78, p = 0.037). The results of regressing the health indices on the date of nesting are presented in **Table 4**. **Table 5** summarizes the results of the regressions of the health parameters on hatching success and emergence success. For the Ca∶P ratio, the coefficient of determination (r^2^) increased from 0.12 to 0.25 (n = 51, p<0.001) when the nests with 0% success (no embryos developing to hatching) are removed from the regression. Similarly, with 0% nests removed, our regression showed that calcium alone significantly correlated with hatching success (n = 52, r^2^ = 0.13, p = 0.009) and emergence success (n = 52, r^2^ = 0.12, p = 0.011).

**Table 1:** Synopsis of plasma protein electrophoretic values for leatherback sea turtles from the literature and this study.

	Florida (this study)			Gabon, Africa^a^			Georgia & Massachusetts, USA^c^		
Parameters	n	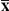 ±SD or median	Range	n	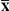 ±SD	Range	n	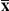 ±SD	Range
Plasma protein (g/dL)	66	3.9±0.6	2.0–5.1	12	N/A	N/A	18	4.6±1.0^d^	2.6–6.2^d^
Pre-albumin (g/dL)	66	0.02	0–0.10	12	0±0	0	N/A	N/A	N/A
Albumin (g/dL)	66	1.45±0.31	0.65–2.19	12	1.18±0.37	1.07–2.39	18	1.76±0.37	0.94–2.44
Alpha (g/dL)	66	0.85	0.32–2.13	12	∼0.98^b^	∼0.58–1.57^b^	18	∼0.92^b^	∼0.13–1.71^b^
Beta (g/dL)	66	0.71	0.18–1.86	12	0.80±0.11	0.60–0.91	18	0.76±0.18	0.41–1.00
Gamma (g/dL)	66	0.67±0.22	0.21–1.32	12	0.81±0.21	0.54–1.28	18	1.52±0.37 (M),1.64±0.22 (F)^e^	0.79–1.91
Albumin∶Globulin	66	0.66	0.25–1.68	N/A	N/A	N/A	NA	N/A	N/A

a Deem et al. (2006); nesting females.

b Reported as alpha-1 and alpha-2 proteins. The two fractions were added for comparative purposes.

c Innis et al. (2010); combined data from directly captured and entangled male and female leatherbacks from Georgia, USA and Massachusetts, USA (not significantly different).

d Total protein determined by a clinical chemistry analyzer, not by refractometry.

e Means of males and females were reported separately, but were not significantly different.

**Table 2:** Synopsis of hematologic values for leatherback sea turtles from the literature (Atlantic Ocean) and this study.

	Florida (this study)			Gabon^c^			Georgia & Massachusetts, USA^d^		
Parameters	n	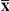 ±SD or Median	Range	n	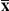 ±SD	Range	n	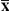 ±SD	Range
PCV^a^ (%)	59	38±4.4	27–50	28	36±5.4	28–56	18	42±7	24–49
RBC^a^^,^ b (×10^3^/µL)	35	362±76	198–515	15	381±198	170–780	N/A	N/A	N/A
WBC^a^^,^ b (×10^3^/µL)	36	2.6±1.0	0.5–4.7	26	4.5±1.6	2.5–7.5	18	8.5±6.0	2.8–22.0
Thrombocytes^b^ (×10^3^/µL)	36	7.6	2.8–22.6	N/A	N/A	N/A	N/A	N/A	N/A
Heterophils^b^ (×10^3^/µL)	24	1.1	0.3–2.6	26	2.4±1.2	0–5.1	18	4.4±2.6	0.5–9.2
Lymphocytes^b^ (×10^3^/µL)	24	1.1	0.2–3.3	26	1.6±0.9	0–3.3	18	2.8±1.5	0.3–5.5
Monocytes^b^ (×10^3^/µL)	24	0.1	0–0.5	26	0.2±0.2	0–0.8	18	0.3±0.3	0–1.0
Eosinophils (×10^3^/µL)	N/A	N/A	N/A	26	0.1±0.1	0–0.5	18^f^	4.0±3.4 (D),0.5±0.7 (E)^f^	0.4–8.9 (D),0.0–1.5 (E)^f^
Basophils (×10^3^/µL)	N/A	N/A	N/A	N/A	N/A	N/A	N/A	0.02±0.05	0–0.2
Heterophils^b^ (%)	40	46.20±15.64	8–80	26^e^	53.33^e^	N/A	18^f^	36±12 (D),60±7 (E)^f^	18–62 (D),48–68 (E)^f^
Lymphocytes^b^ (%)	40	48.15±14.98	20–84	26^e^	35.56^e^	N/A	18	31±14	5–51
Monocytes^b^ (%)	39	5.56±7.65	0–27	26^e^	4.44^e^	N/A	18^h^	See below^f^^,^ g ^,^ h	See below^f^^,^ g ^,^ h
Eosinophils (%)	N/A	N/A	N/A	26^e^	2.22^e^	N/A	18^f^	29±12 (D),7±8 (E)^f^	9–47 (D),1–28 (E)f
Basophils (%)	N/A	N/A	N/A	N/A	N/A	N/A	N/A	0±0	0–1

a PCV = Packed cell volume, RBC = Red blood cells, WBC = White blood cells.

b 2007 season only.

c Deem et al. (2006); nesting females.

d Innis et al. (2010); combined data from directly captured and entangled male and female leatherbacks (not significantly different) from Georgia, USA and Massachusetts, USA.

e Estimated from blood cell counts.

f Significant difference between directly captured (D, n = 11) and entangled (E, n = 7) leatherbacks.

g Significant difference between male and female leatherbacks.

h Monocytes (%) = 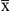
±SD: Directly captured = 2±1 (n = 11), Entangled = 7±8 (n = 7), Male = 1±2 (n = 9), Female = 4±5 (n = 4); Range: Directly captured = 0–5, Entangled = 0–13, Male = 0–5, Female = 1–11.

**Table 3:** Synopsis of plasma biochemical data for leatherback sea turtles from this study (Florida).

Biochemical Test	n	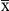 ± SD or Median	Range
ALT (IU/L)^a^^,^ b	7	6	5–20
ALKP (IU/L)^a^	70	31±14	0–76
Amylase (IU/L)^c^	22	1,117±174	794–1,493
Anion gap	46	8.7±4.2	1.1–21.2
AST (IU/L)^a^	69	134	80–355
Bile acids (µmol/L)	69	1	0–26
BUN (mg/dL)^a^	68	1.3	0–3.7
Calcium (mg/dL)	69	10.5±1.8	6.2–15.0
Ca∶P^a^	68	0.85±0.15	0.50–1.20
CO_2_ (mmol/L)	45	29±5	18–40
Chloride (mmol/L)	70	111±4	102–119
Cholesterol (mg/dL)	70	357±54	151–547
CK (IU/L)^a^	70	46	0–6,808
Creatinine (mg/dL)	68	0.1	0–0.2
Glucose (mg/dL)	70	86	43–115
Iron (µg/dL)^c^	23	53	31–93
LDH (IU/L)^a^	48	222	75–987
Lipase (IU/L)^c^	22	21	15–37
Phosphorus (mg/dL)	69	12.3±1.6	7.8–15.9
Potassium (mmol/L)	70	3.9	3.0–11.9
Sodium (mmol/L)	70	144	136–154
Thyroxine (µg/dL)	64	0.6	0–2.2
Total protein (g/dL)	66	4.8±1.1	2.8–7.8
Uric acid (mg/dL)	70	0.4	0–1.3

a ALT = Alanine aminotransferase, ALKP = Alkaline phosphatase, AST = Aspartate aminotransferase, BUN = Blood urea nitrogen, Ca∶P = Calcium∶phosphorus ratio, CK = Creatine kinase, LDH = Lactate dehydrogenase.

b 2008 season only.

c 2007 season only.

**Table 4:** Least-squares linear regression results for health parameters and date of nesting encounter.

Health Parameter	n	Corr^a^ with date	r^2^	p
Albumin	66	−	0.10	0.009
ALKP^a^	69	+	0.10	0.007
Alpha^b^	66	−	0.06	0.044
Anion gap	45	−	0.30	<0.001
BUN^a^^,^ c	68	+	0.13	0.003
CO_2_ a	45	+	0.31	<0.001
Gamma	66	+	0.27	<0.001
Lymphocyte differential	39	−	0.12	0.031
PCV^a^	59	−	0.18	<0.001
RBCs/µL^a^	59	−	0.23	0.004
Thyroxine^c^	64	−	0.08	0.023
Total protein	66	−	0.08	0.025
WBCs/µL^a^	36	−	0.17	0.013

a ALKP = Alkaline phosphatase, BUN = Blood urea nitrogen, CO_2_ = Carbon dioxide, Corr = Correlation, PCV = Packed cell volume, RBC = Red blood cell, WBC = White blood cell.

b Log transformed.

c Rank regression.

**Table 5:** Least-squares linear regression results for health parameters and HS and ES.

Health Parameter	n	Corr with HS^a^	r^2^	p	Corr with ES^a^	r^2^	p
ALKP^a^	56	−	0.09	0.030	−	0.10	0.015
BUN^a^^, ^ b	56	−	0.13	0.007	−	0.07	0.048
Calcium^c^	52	+	0.13	0.009	+	0.12	0.011
Ca∶P^a^	55	+	0.13	0.008	N/A	N/A	N/A
CO_2_ a	33	−	0.29	0.001	−	0.34	<0.001
Cholesterol	57	−	0.16	0.002	−	0.15	0.002
Creatinine^b^	56	N/A	N/A	N/A	+	0.11	0.014
Gamma	54	−	0.10	0.019	−	0.07	0.049
Phosphorus	56	−	0.09	0.024	N/A	N/A	N/A
RBC^a^ count	26	+	0.19	0.031	+	0.29	0.004

a ALKP = Alkaline phosphatase, BUN = Blood urea nitrogen, Ca∶P = Calcium∶phosphorus ratio, CO_2_ = Carbon dioxide, Corr = Correlation, HS = Hatching success, ES = Emergence success, RBC = Red blood cell.

b Using rank regression.

c 0% hatching and emergence success nests removed from statistical tests (subsequently discussed).

## Discussion

This large-scale study provides health parameters of nesting leatherback sea turtles and compares those values to hatching success and emergence success. Our results provide the largest sample size of health indices (plasma protein electrophoresis, hematology, plasma biochemistry) for leatherback turtles. These data add to the previously published health assessments of leatherbacks from Gabon, Africa, Trinidad, Georgia & Massachusetts, USA, Papua New Guinea, Costa Rica, St. Croix, USVI, Equatorial Guinea, and foraging individuals in the Pacific Ocean [1]–[5]. Low hatching success and emergence success have been explored from several nest-based perspectives; however, in Florida's nesting leatherback population, we found that multiple measures of maternal health significantly correlated with leatherback turtle hatching success and emergence success including alkaline phosphatase activity, blood urea nitrogen, calcium, calcium∶phosphorus ratio, carbon dioxide, cholesterol, creatinine, and phosphorus. This and other emerging studies [16], [17] show that the health status of the nesting female or the hatchlings may contribute more to the success of the nest when compared to physical nest dynamics (i.e., water, heat, gases).

### 1. Plasma Protein Electrophoresis

There are a limited number of studies that report plasma protein concentrations in sea turtles [1], [3], [10], [31], [32] and few, if any, attempt to explain the data obtained, making interpretation of results difficult. In our study, all but 2 turtles had protein electrophoretic values that fell within the normal reported range (3–7 g/dL) for reptiles [9]. Total protein concentrations in nesting females from Florida were similar to concentrations observed in nesting females from Gabon, Africa [1] and slightly lower (by ∼0.7 g/dL on average) than concentrations found in leatherbacks from northwestern Atlantic Ocean foraging grounds [3]. Florida's nesting leatherbacks had lower (by ∼0.4 g/dL on average) total protein concentrations than adult, wild-caught male and female loggerhead sea turtles (*Caretta caretta*) from Florida Bay [31]. The lower concentrations of protein found in nesting females when compared to males and non-nesting females indicate that gravid leatherbacks may experience protein loss during the nesting season. This is further verified by the observed decrease in proteins (decrease in total protein and albumin, **Table 4**) across the nesting season reported in our study. This trend is also common in birds, where total protein decreases during egg production [33]. Honarvar et al. [5] observed a decrease in albumin across the season in nesting leatherbacks. They attributed this to nutritional stress, disease, decreased protein production, or depletion of bodily reserves. Deem et al. [32] found no significant differences between total protein concentrations in nesting female loggerheads and foraging loggerheads from Georgia; however, Harris et al. [4] found significantly lower total protein concentrations in nesting leatherbacks from Papua New Guinea, Costa Rica, and St. Croix when compared to individuals foraging off the coast of California. Direct comparisons with our values should not be made because Harris et al. [4] measured total protein with a plasma biochemistry analyzer, which calculates concentrations using proprietary formulas based upon mammalian blood. Our values were determined by direct electrophoretic measurement [34], [35]. Anorexia may at least partially explain the lower protein concentrations of the two turtles from our study [36], as prey consumption is generally low during the nesting season [37].

Leatherbacks from our study had concentrations of albumin that were slightly higher (by ∼0.27 g/dL on average) than nesting leatherbacks from Gabon, Africa [1] and slightly lower (by ∼0.31 g/dL on average) than leatherbacks (free-ranging or disentangled from fishing gear) from Georgia and Massachusetts, USA [3]. Decreased albumin concentrations in nesting leatherbacks could be consistent with egg formation, although the differences we observed are slight. Deem et al. [32] found higher concentrations of albumin in nesting loggerheads than foraging loggerheads. The total alpha and beta globulin (inflammatory-responsive acute-phase proteins; i.e., alpha_1_-antitrypsin, alpha_2_-macroglobulin, beta-lipoprotein, ceruloplasmin, fibrinogen, haptoglobin, transferrin) [31], [35], [38] concentrations in leatherbacks from our study were similar to the Gabon, Africa and Georgia and Massachusetts studies [1], [3]. Gamma globulins (i.e., pathogen-eliminating immunoglobulins) [31], [33], [35] were lower (∼0.85–0.97 g/dL on average) in nesting females when compared to leatherbacks off the coasts of Georgia and Massachusetts [1], [3]. As of now, this finding is unclear, but could be related to immune responses of foraging individuals [3]. Lower concentrations of globulins may also be a result of decreased food intake of nesting females. Lastly, leatherbacks from this and other studies tended to have higher albumin∶globulin ratios than loggerheads, but a ratio similar to that found in green turtles (*Chelonia mydas*) [1], [3], [10], [31], [32]. This higher ratio could be attributed to differences in diet (loggerheads are carnivorous, often feeding on mollusks and crabs; green turtles are herbivorous as adults; leatherbacks consume gelatinous zooplankton consisting of jellyfish, salps, and pyrosomes) [39]. The leatherback and loggerhead diet drastically differ, and because the prey items of leatherbacks are composed of ∼95% saltwater [40] and leatherbacks are known to consume high prey volumes daily [41], [42], albumin concentrations may be elevated, caused by dehydration due to the high intake of salt [36]. The increased ratio is a result of generally higher albumin concentrations in green turtles and leatherbacks and similar concentrations of globulin when compared to loggerheads [1], [3], [10], [31], [32]. It has been suggested that hypoalbuminemia is a physiological adaption to prolonged dives that aquatic turtles often make [43]. Low albumin concentrations may be associated with the accumulation of coelomic fluid (ascites) that has a high concentration of bicarbonate, which acts as a buffer against lactic acidosis (e.g., associated with deep dives) [31], [43]. Although leatherbacks dive deeper on average, loggerheads make longer routine dives and spend more time submerged [44], possibly explaining the lower albumin concentrations in loggerheads compared to leatherbacks.

We found that the gamma globulin protein was negatively correlated with both hatching success and emergence success, a finding similar to that observed in collared flycatchers (*Ficedula albicollis*) [45]. Gamma globulins consist of the immunoglobulins [31], [33] and increased concentrations of this plasma protein may be related to chronic inflammatory, infectious, and immune-mediated diseases in birds [46], [47]. Additionally, their immune system may be suppressed due to stress induced by nesting [47], which is validated by the increase in the gamma globulin protein across the season, as was observed in our study (**Table 4**). The immune system of the nesting females with increased concentrations of gamma globulins may be responding to disease agents (i.e., bacteria, viruses, etc.). These agents may be transferred from nesting female to offspring via the egg yolk or egg components, which has been shown to occur in avian eggs [48]. Most of our protein fractions fell within normal ranges for reptiles [9], suggesting that nesting leatherbacks from Florida likely are not experiencing hypo- or hyperproteinemia. The increased concentrations of immunoglobulins in nesting leatherbacks may be associated with egg production, as these are known to be transported into the oocytes for uptake by developing chick embryos [49], [50]. Our data point to the need for studies focused upon resolving the relationships between the gamma globulin protein and hatching success and/or emergence success to determine if the trend is common or spurious.

### 2. Hematology

Leatherbacks have the highest PCV of any reptile, which is surmised to be an adaptation to their increased oxygen requirement for making frequent and prolonged dives [51]. The PCV of the leatherbacks in our study was similar to reports from other studies of nesting female leatherbacks from Gabon, Costa Rica, Papua New Guinea, and St. Croix [1], [4], [51] and to non-reproductive male and female leatherbacks captured off the coasts of Georgia and Massachusetts [3]. Packed cell volume was much lower in Atlantic Ocean and Pacific Ocean basin nesting females [1], [4], [5, this study] compared to foraging individuals off the coast of California. This difference could be related to nutritional differences of Atlantic and Pacific leatherbacks [4]. Additionally, Pacific leatherbacks generally make much longer migrations than Atlantic leatherbacks [52]. Longer migrations could lead to dehydration, which would increase PCV. Published studies found no differences in PCV between non-nesting female and male leatherback turtles [3], [4].

Leukocyte differentials for the nesting females in our study fell within the normal range for reptiles [36], [53]. We found a slightly higher percentage of lymphocytes than heterophils in our study, which is normal, as most reptiles have more circulating lymphocytes than any other leukocyte [54]. Within our Florida nesting population, the total leukocyte count decreased as the nesting season progressed, typical of a stress leukogram (result of systemic stress, decrease in lymphocytes, increase in monocytes). Nesting leatherbacks lay multiple clutches during the nesting season and travel 200–700 km between subsequent nesting events before returning to the nesting beach [55]. As the season progresses, nesting females may become more stressed and fatigued (as is seen in migratory loggerheads) [56], resulting in this type of leukogram. It is important to note that there is difficulty in comparing blood cell counts and differentials from other studies as there likely are differences in methodologies. Dissimilar blood cell count methods may account for some of the variation across studies reported here [1], [3], [4] (**Table 2** **, Table S1**). A standardized method should be established for at least marine turtle hematologic measures.

Thrombocyte counts have not previously been described for leatherbacks; other studies have evaluated their functions, which are similar to bird and mammalian platelets [36], [57], [58]. Few studies report thrombocyte counts in sea turtles in general. The leatherbacks' thrombocyte counts from this study were greater than those of green turtles from the Cayman Turtle Farm [59]. The number of thrombocytes in leatherbacks from our study is on the high end of the normal range for reptiles [60]. Thrombocyte aggregation in vertebrates occurs during decompression [61]. In order to avoid depletion of thrombocytes with these frequent clots, leatherbacks may produce a greater number due to their numerous and prolonged dives.

We found that nesting females with higher total erythrocyte counts produced nests with higher hatching success and emergence success. This finding is significant, as a lower erythrocyte count may be indicative of anemia of chronic disease. This is to say that any chronic, although non-debilitating condition tends to cause anemia [32]. Deem et al. [32] found that stranded loggerhead turtles had significantly lower PCVs compared to foraging and nesting individuals (PCV and total erythrocyte count were significantly correlated in our study). The authors attributed low PCV to the chronic state of stress in the turtles (i.e., poor nutrition, infectious disease, and/or immune deficiency). If nesting females are mildly anemic due to a chronic condition, conceivably the fate of their nests could be adversely affected. Therefore, they may provision their eggs with insufficient nutrient or mineral concentrations needed for the embryos to survive. In Uppsala, Sweden, great tits (*Parus major*) with decreased PCV produced clutches with fewer eggs, which may be related to the health of the nesting individuals. Additionally, those birds were unable to produce large eggs, affecting the survival of the offspring [62]. The PCV values reported for the nesting females are lower than those observed in foraging individuals [4]; however, they still fall within the normal range for this species and indicate that anemia is not present. Our findings suggest that further research is warranted to understand the functional relationships leading to such results.

We found that hatching success and emergence success of nests laid earlier in the nesting season were higher than those laid later in the season. Packed cell volume and erythrocyte count were higher at the beginning of the nesting season than the end. These findings indicate that females laying at the beginning of the nesting season could be in better physical condition than at the end of the nesting season. These findings are similar to those of birds that arrive early to breeding and nesting grounds. Early arrival is associated with more robust physiological condition (i.e., higher PCV, higher fat scores, lower lymphocyte counts) [63], [64]. Theoretically, a large portion of the reproductive leatherback population may characteristically experience mild, chronic anemia, which may lead to the production of less successful nests. If anemia is present in the leatherback population, it most likely would not be related to dietary deficiencies (i.e., iron), as jellyfish have high concentrations of iron in their tissues [65]. Since few reports of blood parameters for leatherbacks exist [1]–[5, our study], we cannot be sure that those with persistent anemia or chronic low-level disease are not incorporated into health indices from free-ranging populations, particularly if low-level disease is common. The observed correlations between higher gamma globulins and lower RBC counts with lower hatching success and emergence success provides speculation that less healthy individuals may produce less successful nests.

### 3. Plasma Biochemistry

Sodium and chloride concentrations in nesting leatherbacks from Florida were comparable to other published values for nesting leatherbacks [1], [2], [4], [5]. Additionally, nesting female sea turtles appear to have lower sodium and chloride concentrations than directly captured individuals and foraging individuals [3], [4], [32]. The lower values found in nesting females are most likely due to reduced food intake during the nesting season [37]. Harris et al. [4] found differences in sodium concentrations between Atlantic leatherbacks (St. Croix) and Pacific leatherbacks (Costa Rica, Papua New Guinea), indicating possible dietary differences. Our values for potassium fell within published ranges for reptiles [36]. However, Pacific foraging leatherbacks and Atlantic foraging loggerheads had higher concentrations of potassium than nesting individuals. This is most likely explained by decreased food intake by nesting sea turtles [4], [32].

We observed a significant increase in the anion gap and a significant decrease in CO_2_ as the season progressed. However, we did not measure for CO_2_ immediately following collection, which may have skewed our results. Therefore, we cannot make any assumptions about the anion gap or maternal CO_2_ concentrations and the negative correlation observed with hatching success and emergence success.

Calcium and phosphorus concentrations from Florida's nesting leatherbacks were similar to values reported for other leatherbacks [1]–[5]. Leatherbacks tend to have a low Ca∶P ratio (<1) when compared to other reptiles and sea turtles possibly due to their diet [1]–[5], [9], [66]–[68], although maturity status could have an effect on this ratio (adult sea turtles may have a lower ratio in comparison to juveniles, as juveniles absorb calcium more efficiently, resulting in higher calcium concentrations and a higher ratio). Calcium concentrations in leatherbacks in our study significantly decreased in subsequent sampling events. Vitellogenesis is most likely complete prior to the nesting season [22], [23]; however, residual calcium may be present in the nesting females earlier in the season as a result of this process. Rostal et al. [22], [23] found that calcium concentrations in leatherbacks were relatively constant during the nesting season, but they observed that calcium decreased slightly from the beginning to the end of the nesting season. A decline in calcium concentrations during the nesting season was also observed in Kemp's ridley sea turtles (*Lepidochelys kempii*) [69] and nesting leatherback sea turtles from Equatorial Guinea [5]. Honarvar et al. [5] suggest that this decrease signifies a return to calcium concentrations that are representative of the species before folliculogenesis commences. This trend is similar to calcium concentrations in nesting Kemp's ridleys from the Cayman Turtle Farm [69].

We found that the Ca∶P ratio positively correlated with hatching success of leatherback sea turtles from Florida. The Ca∶P ratio of prelaying greater sage grouse (*Centrocercus urophasianus*) was found to be important in chick survival and the authors suggest that a balance calcium and phosphorus is needed, or egg production and offspring survival can be affected [70]. By considering only nests that had the potential to produce hatchlings (omitted those that failed to develop at all, likely due to physical environmental factors), the regressions showed that calcium alone significantly affected hatching success and emergence success. These results are similar to those found in a study of great tits from the Netherlands, which found that individuals feeding in areas with calcium-deficient soils produced thin and porous-shelled eggs. Those individuals also produced nests with hatching success rates that were 15–25% lower than those from birds that fed on more calcium-rich diets [71]. Additionally, we found that those nesting females with higher concentrations of phosphorus produced nests with lower hatching success. High plasma phosphorus content may negatively affect the quality of the eggshell by increasing preservation of calcium in order to maintain the Ca∶P ratio, thereby decreasing the amount of calcium allotted for eggshell formation [72].

Creatine kinase activity was variable within the Florida leatherback population (0–6,808 IU/L); this is consistent with findings in other leatherback studies [1]–[5]. Some individuals with obvious external injuries exhibited higher (>1000 IU/L) CK activity. Elevated activities of CK, AST and LDH can also result from hemolysis or repeated venipuncture attempts immediately prior to sampling [36], which may explain the higher concentrations in some of the individuals.

Alanine aminotransferase (ALT) activity was in the normal range for reptiles [36] and similar to other published values for leatherbacks [1], [3], [5]. Alkaline phosphatase activity (ALKP) was negatively correlated with hatching success and emergence success of leatherbacks from our study. This enzyme is not tissue specific in reptiles, making interpretation of this finding difficult. Campbell [36] states that high activity of ALKP may be related to an increase in osteoblastic activity. Those turtles with higher ALKP activity may be retaining calcium (for bone formation) and depositing less into the eggshells. Because reproduction is a “luxury,” these organisms may be differentially rebuilding bone over depositing calcium into the eggshell, leading to a lower hatching success and emergence success. Alkaline phosphatase activity increased as the nesting season progressed, indicating that bone-formation may up-regulate towards the end of the nesting season. This trend needs further exploration to determine if it is biologically significant or a spurious relationship.

Blood urea nitrogen (BUN) and uric acid concentrations in nesting leatherbacks and loggerheads are low compared to foraging individuals, indicating that food intake is reduced during the nesting season [1]–[5], [32]. This makes the observed increase in BUN across the nesting season puzzling (**Table 4**), but it may be related to dehydration. Blood urea nitrogen and creatinine were negatively correlated with hatching success and emergence success; however, BUN and creatinine are thought to be of poor diagnostic value to reptiles [36], [73] and most likely the trends observed here are spurious.

Glucose concentrations in leatherbacks are similar in the different populations and across ocean basins [1]–[5], implying that this biochemical parameter regulates toward a homeostatic concentration. Harris et al. [4] found that foraging individuals and nesting individuals showed no differences in glucose concentrations, which suggests that these levels remain constant in clinically healthy turtles, regardless of reproductive state. However, the glucose concentrations in nesting leatherbacks from Papua New Guinea and Costa Rica (Pacific) averaged slightly higher (∼10–20 mg/dL on average) than concentrations previously published for Atlantic leatherbacks [1]–[5]. It is conceivable that the concentrations are due to prey in different ocean basins exhibiting differing energy densities.

Activities of amylase and lipase were higher in nesting leatherbacks from Florida compared to those reported from Gabon [1] and Equatorial Guinea [5]. Honarvar et al. [5] suggested that higher amylase activity observed in leatherbacks from Gabon when compared to Equatorial Guinea's nesting population may reflect opportunistic feeding during the nesting season. Additionally, different populations may have distinct enzyme activities. For example, alligator snapping turtles from Florida and Georgia significantly differed in their amylase and lipase activities based on locale [74]. Leatherbacks also have higher lipase activity than loggerheads from Georgia [32] and Kemp's ridleys from the New York bight [75], most likely associated with the extensive fatty tissues of leatherbacks [14], [76]. Lastly, nesting female loggerheads had higher lipase activity than foraging individuals [32]. We hypothesize that this may be related to follicular atresia, which would also increase their bodily lipase activity. Reabsorption of follicles may provide nesting females with an alternate energy source during the nesting season [77], [78] to meet their metabolic demands.

Cholesterol concentrations in leatherbacks appear to be elevated in comparison to those of loggerhead and green turtles [1], [3], [4], [10], [32], [79]–[82]. Loggerheads should have higher cholesterol than leatherbacks, as the leatherback diet is low in cholesterol [83], whereas the loggerhead diet is not. The high body fat of leatherbacks, in comparison to the other sea turtle species, may contribute to the higher cholesterol concentrations in leatherbacks. The negative correlations we found between maternal cholesterol and hatching success and emergence success are puzzling, as cholesterol is important in developing reptilian embryos [84]. However, high cholesterol can be linked to liver damage and renal protein loss [85]. Hepatic impairment and protein deficiency may lead to suboptimal yolk production, leaving the embryo with an insufficient quantity or quality of nutrients for proper development. It is interesting to note that those individuals that produced nests with 0% hatching and emergence success had cholesterol concentrations that were 120 mg/dL higher, on average, than those that had successful nests (>0%).These diverse potential explanations suggest several avenues of further exploration.

To our knowledge, iron concentrations have not been reported in leatherback turtles. Plasma iron concentrations have been previously published for nesting hawksbill (*Eretmochelys imbricata*) and olive ridley (*Lepidochelys olivacea*) sea turtles, wild juvenile, subadult and adult green turtles from Australia, Hawaii, the Bahamas, and the United Arab Emirates [67], [79], [80], [82], [86]. Iron concentrations in male and female adult green turtles from Australia and the United Arab Emirates were similar to our findings. Nesting hawksbills and olive ridleys from Oman also had similar iron concentrations to nesting leatherbacks from our study [86]. Rarely do those studies that report iron concentrations discuss the results. Hasbún et al. [79] found that larger turtles had higher iron concentrations than smaller turtles and that females had significantly higher iron concentrations than males. Stoneburger et al. [87] and Sakai et al. [88] found high concentrations of iron in the yolk of loggerhead and green sea turtle eggs. Storelli et al. [89] found the highest concentrations of iron in the liver of sea turtles compared to other tissues, suggesting that the liver (as well as the spleen) acts as a storage organ for iron in sea turtles, which is similar to domestic chickens (*Gallus gallus*) [90]. As females prepare their follicles, iron may be transferred to the yolk at high concentrations (yolk is formed through hepatic processes) to support normal embryonic development. Iron in the plasma of hens significantly increased shortly before egg-laying; after egg production was complete, iron concentrations returned to normal [91]. Nesting females' iron concentrations may be elevated during folliculogenesis, as has been observed in alligators (*Alligator mississipiensis*) [92], so that they may pass on adequate amounts to their offspring. Data on wild-caught or foraging individuals are needed for leatherbacks to determine if concentrations of iron in the nesting females from our study are representative for this species or if they reflect normal concentrations for reproductive animals.

No previous reports of thyroxine concentrations in leatherback turtles exist. The concentrations in Florida's leatherbacks were similar to those reported for nesting Kemp's ridley sea turtles [69]. In sea turtles and other marine species, thyroxine may be related to nutrient mobilization required during vitellogenesis and for metabolic stimulation during mating [93]. Thyroxine decreased across the nesting season within Florida's turtles, which is consistent with the hypothesis that it is important during vitellogenesis. Elevated concentrations of thyroxine following vitellogenesis may remain at the beginning of the nesting season and then may taper off at its close. As with other health parameters, thyroxine values for non-nesting leatherbacks are needed for more accurate interpretation.

### 4. Conclusions

Our study is the first to correlate maternal health parameters with reproductive success in sea turtles. Although many of our coefficient's of determination (r^2^) were low, it is noteworthy that the nests in our study were left *in situ* and were subject to many biotic and abiotic factors that also could have affected hatching success and emergence success. Therefore, both maternal health and environmental factors are important to leatherback turtle reproductive success. Because population differences most likely exist, our results provide valuable measures of health indices for future examination of this population, comparisons with other populations, and with other sympatric turtle species. Infectious diseases are among the most important problems influencing the survival rates of animals, particularly wildlife; therefore, it is essential that such problems are characterized. Our data demonstrate intriguing patterns across individuals and allow us further understanding into egg and hatchling mortality which comprise nest failure. Our study will allow biologists and veterinarians to (i) better monitor wild populations, (ii) assist managers to understand challenges to productivity, (iii) help veterinarians to better treat sick animals and improve on their interpretations of laboratory results, and (iv) aid pathologists in diagnosing causes of illness or death.

## Supporting Information

Table S1 Synopsis of hematologic values for leatherback sea turtles from the literature (Pacific Ocean).
(DOC)

Table S2 Synopsis of plasma biochemical data for leatherback sea turtles from the literature (western Atlantic Ocean).
(DOC)

Table S3 Synopsis of plasma biochemical data for leatherback sea turtles from the literature (eastern Atlantic Ocean).
(DOC)

Table S4 Synopsis of plasma biochemical data for leatherback sea turtles from the literature (Pacific Ocean).
(DOC)
