# Supplementary material for: Maternal Health Status Correlates with Nest Success of Leatherback Sea Turtles (Dermochelys coriacea) from Florida
Source: PLoS One. 2012 Feb 16;7(2):e31841. doi: 10.1371/journal.pone.0031841 (PMC3281022; doi:10.1371/journal.pone.0031841)
Supplement: Table S1 — Synopsis of hematologic values for leatherback sea turtles from the literature (Pacific Ocean). (DOC) [file pone.0031841.s001.doc]

Supplemental Table S1. Synopsis of hematologic values for leatherback sea turtles from the literature (Pacific Ocean).

|  | **California, Foraging Females^b^** | | | **California, Foraging Males^b^** | | | **Costa Rica & Papua New Guinea^b,c^** | | |
| --- | --- | --- | --- | --- | --- | --- | --- | --- | --- |
| **Parameters** | **n** | **Median** | **Range** | **n** | **Median** | **Range** | **n** | **Median** | **Range** |
| PCV (%) | 7 | 54^d^ | 48-63 | 5 | 57 | 50-71 | 17 | 37 | 30-45 |
| RBC (x 10^3^/µL) | 7 | 720^d^ | 340-1060 | 5 | 580 | 470-930 | 17 | 380 | 140-590 |
| WBC (x 10^3^/µL) | 7 | 12.3^d^ | 9.0-20.3 | 5 | 12.3 | 8.3-16.0 | 17 | 8.4 | 4.5-9.5 |
| Heterophils (x 10^3^/µL) | 7 | 7.4^d^ | 4.1-9.1^c^ | 5 | 7.4 | 5.9-9.1 | 17 | 2.6 | 1.4-5.6 |
| Lymphocytes (x 10^3^/µL) | 7 | 3.9^d^ | 1.8-6.2^c^ | 5 | 2.6 | 1.8-6.2^c^ | 17 | 4.1 | 2.3-6.6 |
| Monocytes (x 10^3^/µL) | 7 | 0.5^d^ | 0-1.3^c^ | 5 | 0.2 | 0.1-0.5 | 17 | 0.1 | 0-0.3 |
| Eosinophils (x 10^3^/µL) | 7 | 1.6 | 0-6.5^c^ | 5 | 0.9 | 0.1-2.7 | 17 | 0.1 | 0-0.4 |
| Basophils (x10^3^/ µL) | N/A | N/A | N/A | N/A | N/A | N/A | N/A | N/A | N/A |
| Heterophils^b^ (%) | 7 | 60.00^e^ | N/A | 5 | 59.84^e^ | N/A | 17 | 30.74^e^ | N/A |
| Lymphocytes^b^ (%) | 7 | 31.36^e^ | N/A | 5 | 20.74^e^ | N/A | 17 | 49.17^e^ | N/A |
| Monocytes^b^ (%) | 7 | 3.66^e^ | N/A | 5 | 1.19^e^ | N/A | 17 | 0.81^e^ | N/A |
| Eosinophils (%) | 7 | 13.12^e^ | N/A | 5 | 7.30^e^ | N/A | 17 | 1.02^e^ | N/A |
| Basophils (%) | N/A | N/A | N/A | N/A | N/A | N/A | N/A | N/A | N/A |
| ^a^ PCV = Packed cell volume, RBC = Red blood cells, WBC = White blood cells  ^b^ Harris et al. (2011), leatherbacks from Pacific Costa Rica and Papua New Guinea were nesting individuals  ^c^ No significant differences in hematology between nesting leatherbacks from Costa Rica and nesting leatherbacks from Papua New Guinea  ^d^ Significant difference between foraging leatherbacks and nesting female leatherbacks  ^e^ Estimated from blood cell counts | | | | | | | | | |
