# Supplementary material for: Maternal Health Status Correlates with Nest Success of Leatherback Sea Turtles (Dermochelys coriacea) from Florida
Source: PLoS One. 2012 Feb 16;7(2):e31841. doi: 10.1371/journal.pone.0031841 (PMC3281022; doi:10.1371/journal.pone.0031841)
Supplement: Table S2 — Synopsis of plasma biochemical data for leatherback sea turtles from the literature (western Atlantic Ocean). (DOC) [file pone.0031841.s002.doc]

Supplemental Table S2. Synopsis of plasma biochemical data for leatherback sea turtles from the literature (western Atlantic

Ocean).

|  | **St. Croix^b^** | | | **Trinidad^c^** | | | **Georgia & Massachusetts, USA^d^** | | |
| --- | --- | --- | --- | --- | --- | --- | --- | --- | --- |
| **Biochemical Test** | **n** | **Median** | **Range** | **n** | $\bar{x}$**± SD** | **Range** | **n** | $\bar{x}$**± SD** | **Range** |
| ALT (IU/L) ^a^ | N/A | N/A | N/A | N/A | N/A | N/A | 18 | 11±10 | 0-44 |
| ALKP (IU/L)^a^ | 12 | 41 | 27-61 | N/A | N/A | N/A | 18 | 85±54 | 13-199 |
| Amylase (IU/L) | N/A | N/A | N/A | N/A | N/A | N/A | N/A | N/A | N/A |
| Anion gap | N/A | N/A | N/A | 13 | 9±3 | 1-14 | 7 (M),  3 (F)^a,f^ | 13.3±3.0 (M),  11.1±6.0 (F)^a,f^ | 7.1-18.5^f^ |
| AST (IU/L)^a^ | 12 | 100 | 71-153 | 13 | 191±83 | 100-365 | 18 | 286±269 | 97-1,312 |
| Bile acids (µmol/L) | N/A | N/A | N/A | N/A | N/A | N/A | 11 | 5±5 | 0-16 |
| BUN (mg/dL)^a^ | N/A | N/A | N/A | 13 | 1.1±0.3^e^ | 1.7-4.8^e^ | 11 (DC),  7 (E)^a,g^ | 128±29 (DC),  83±31 (E)^a,g^ | 40-174^g^ |
| Calcium (mg/dL) | 12 | 9.9 | 7.4-14.4 | 13 | 13.2±2.1 | 10.1-16.8 | 18 | 6.0±1.7 | 1.8-9.3 |
| Ca:P^a^ | 12 | ~0.86 | N/A | 13 | ~0.61 | N/A | 18 | ~0.67 | ~0.28-0.80 |
| CO_2_ (mmHg) | N/A | N/A | N/A | 13 | 38±4 | 35-45 | 15 | 35±5 | 23-43 |
| Chloride (mmol/L) | N/A | N/A | N/A | 13 | 109±3 | 104-117 | 11 (DC),  7 (E)^a,g^ | 121±5 (DC),  113±7 (E)^a,g^ | 102-130^g^ |
| Cholesterol (mg/dL) | 12 | 341 | 195-454 | N/A | N/A | N/A | 18 | 285±79 | 142-438 |
| CK (IU/L)^a^ | 12 | 44 | 8-5,139 | 13 | 711±435 | 36-1,385 | 18 | 1,388±3,092 | 40-13,262 |
| Creatinine (mg/dL) | N/A | N/A | N/A | N/A | N/A | N/A | 18 | 0.4±0.5 | 0-1.7 |
| Glucose (mg/dL) | 12 | 82 | 64-105 | 13 | 83±9 | 72-103 | 18 | 87±21 | 58-130 |
| Iron (µg/dL) | N/A | N/A | N/A | N/A | N/A | N/A | N/A | N/A | N/A |
| LDH (IU/L)^a^ | 12 | 456 | 297-1,351 | N/A | N/A | N/A | 18 | 649±375 | 276-1,632 |
| Lipase (IU/L) | N/A | N/A | N/A | N/A | N/A | N/A | N/A | N/A | N/A |
| Phosphorus (mg/dL) | 12 | 12.6 | 10.6-15.4 | 13 | 16.6±2.2 | 13.1-19.9 | 18 | 9.0±1.7 | 6.5-11.6 |
| Potassium (mmol/L) | 12 | 3.6 | 2.9-4.7 | 13 | 3.9±0.9 | 3.0-5.8 | 18 | 5.0±0.9 | 3.7-7.0 |
| Sodium (mmol/L) | 12 | 141 | 135-149 | 13 | 143±3 | 139-150 | See below^g,h,i^ | See below^g,h,i^ | See below^g,h,i^ |
| Total protein (g/dL) | 12 | 4.1 | 2.8-4.7 | 13 | 4.9±0.6 | 4.1-6.2 | 18 | 4.6±1.0 | 2.6-6.2 |
| Uric acid (mg/dL) | 12 | 0.4 | 0.3-0.5 | N/A | N/A | N/A | 11 | 1.3±0.8 | 0.5-3.9 |
| ^a^ ALT = Alanine aminotransferase, ALKP = Alkaline phosphatase, AST = Aspartate aminotransferase, BUN = Blood urea nitrogen, Ca:P = Calcium:phosphorus ratio, CK = Creatine kinase, DC = Directly captured, E = Entangled, LDH = Lactate dehydrogenase  ^b^ Harris et al. (2011), nesting females  ^c^ Harms et al. (2007); nesting females  ^d^ Innis et al. (2010); combined data from directly captured and entangled male and female leatherbacks  ^e^ Mean reported is less than the lowest value of the range  ^f^ Means of males and females were reported separately, but were not significantly different; ranges were combined for this table  ^g^ Significant difference between directly captured and entangled leatherbacks; ranges were combined for this table  ^h^ Significant difference between male and female leatherbacks; ranges were combined for this table  ^i^ Sodium (mmol/L)= $\bar{x}$±SD: Directly captured = 157±3 (n = 11), Entangled = 154±5 (n = 7), Male = 157±3 (n = 9), Female = 153±6 (n = 4); Range: Directly captured = 153-161, Entangled = 145-162, Male = 155-162, Female = 145-158 | | | | | | | | | |
