# Supplementary material for: Maternal Health Status Correlates with Nest Success of Leatherback Sea Turtles (Dermochelys coriacea) from Florida
Source: PLoS One. 2012 Feb 16;7(2):e31841. doi: 10.1371/journal.pone.0031841 (PMC3281022; doi:10.1371/journal.pone.0031841)
Supplement: Table S3 — Synopsis of plasma biochemical data for leatherback sea turtles from the literature (eastern Atlantic Ocean). (DOC) [file pone.0031841.s003.doc]

Supplemental Table S3. Synopsis of plasma biochemical data for leatherback sea turtles from the literature (eastern Atlantic Ocean).

|  | **Equatorial Guinea^b^** | | | | **Gabon^f^** | | | |
| --- | --- | --- | --- | --- | --- | --- | --- | --- |
| **Biochemical Test** | **n^b^** | $\bar{x}$**± SD^c^** | $\bar{x}$**± SD^d^** | **Range^b^** | **n^f^** | $\bar{x}$**± SD^g^** | $\bar{x}$**± SD^h^** | **Range^f^** |
| ALT (IU/L) ^a^ | 18 | 9±0 | 9±0 | 3-15 | 17 | 4±1 | 4±2 | 3-10 |
| ALKP (IU/L)^a^ | 20 | 52±2 | 38±2 | 30-75 | N/A | N/A | N/A | N/A |
| Amylase (IU/L) | 20 | 346±9 | 291±12 | 215-477 | 18 | 681±104 | 628±71 | 495-895 |
| AST (IU/L)^a^ | 55 | 126±3 | 129±7 | 78-174 | 18 | 165±29 | 159±49 | 94-234 |
| Bile acids (µmol/L) | 20^e^ | 4±1^e^ | 3±0^e^ | 3-9^e^ | N/A | N/A | N/A | N/A |
| BUN (mg/dL)^a^ | 6 | 2.3±0.1 | 2.0 | 1.3-3.3 | 17 | 2±0 | 3±4 | 2-13 |
| Calcium (mg/dL) | 55 | 11.1±0.3 | 11.3±0.6 | 7.5-14.8 | 18 | 8±2 | 7.1±1.8 | 4.4-10.0 |
| Ca:P^a^ | 55 | ~0.92 | ~0.94 | N/A | 18 | ~0.65 | ~0.73 | N/A |
| CO_2_ (mmol/L) | N/A | N/A | N/A | N/A | 17 | 23±2 | 22±2 | 18-25 |
| Cholesterol (mg/dL) | N/A | N/A | N/A | N/A | 18 | 293±73 | 346±96 | 136-497 |
| CK (IU/L)^a^ | 52 | 147±21 | 151±35 | N/A-453 | 18 | 287±359 | 1,228±2,390 | 20-7,086 |
| Creatinine (mg/dL) | N/A | N/A | N/A | N/A | 17 | 0.2±0.1 | 0.3±0.1 | 0.1-0.5 |
| Glucose (mg/dL) | 55 | 86±2 | 86±3 | 60-113 | 18 | 84±9 | 78±13 | 55-95 |
| LDH (IU/L)^a^ | N/A | N/A | N/A | N/A | 18 | 1,502±528 | 1,716±852 | 793-3,564 |
| Lipase (IU/L) | N/A | N/A | N/A | N/A | 18 | 1±0 | 2±2 | 1-6 |
| Phosphorus (mg/dL) | 55 | 12.2±0.2 | 12.0±0.3 | 9.9-14.5 | 17 | 11.0±2.0 | 11.0±1.5 | 8.9-14.0 |
| Potassium (mmol/L) | 55 | 4.1±0.1 | 4.4±0.1 | 3.3-4.9 | 17 | 4.0±1.0 | 4.0±0.9 | 2.8-5.1 |
| Sodium (mmol/L) | 55 | 140±0 | 140±1 | 135-146 | 17 | 136±9 | 138±6 | 124-148 |
| Total protein (g/dL) | 54 | 5.1±0.1 | 5.0±0.2 | 3.6-6.6 | 18 | 4.0±1.0 | 4.6±1.0 | 3.0-6.0 |
| Uric acid (mg/dL) | 45 | 0.4±0.0 | 0.4±0.0 | 0.2-0.6 | 18 | 0.2±0.0 | 0.2±0.0 | 0.2 |
| ^a^ ALT = Alanine aminotransferase, ALKP = Alkaline phosphatase, AST = Aspartate aminotransferase, BUN = Blood urea nitrogen, Ca:P = Calcium:phosphorus ratio, CK = Creatine kinase, LDH = Lactate dehydrogenase  ^b^  Honarvar et al. (2011), nesting females, n and range reported are combined for all sampled leatherbacks  ^c^ All sampled leatherbacks  ^d^ First-time nesters  ^e^ Total bilirubin  ^f^ Deem et al. (2006), nesting females, n and range reported are combined from blood collected in sodium heparin and lithium heparin tubes (no values were statistically different)  ^g^ Samples collected in lithium heparin  ^h^ Samples collected in sodium heparin | | | | | | | | |
