# Supplementary material for: Maternal Health Status Correlates with Nest Success of Leatherback Sea Turtles (Dermochelys coriacea) from Florida
Source: PLoS One. 2012 Feb 16;7(2):e31841. doi: 10.1371/journal.pone.0031841 (PMC3281022; doi:10.1371/journal.pone.0031841)
Supplement: Table S4 — Synopsis of plasma biochemical data for leatherback sea turtles from the literature (Pacific Ocean). (DOC) [file pone.0031841.s004.doc]

Supplemental Table S4. Synopsis of plasma biochemical data for leatherback sea turtles from the literature (Pacific Ocean).

|  | **California, Foraging Females^b^** | | | **California, Foraging Males^b^** | | | **Costa Rica, Nesting Females^b^** | | | **Papua New Guinea, Nesting Females^b^** | | |
| --- | --- | --- | --- | --- | --- | --- | --- | --- | --- | --- | --- | --- |
| **Biochemical Test** | **n** | **Median** | **Range** | **n** | **Median** | **Range** | **n** | **Median** | **Range** | **n** | **Median** | **Range** |
| ALKP (IU/L)^a^ | 9 | 79^c^ | 50-290 | 5 | 817 | 80-4,098 | 8 | 40^d^ | 25-53 | 11 | 61^d^ | 36-240 |
| AST (IU/L)^a^ | 9 | 165^c^ | 111-731 | 5 | 166 | 121-185 | 8 | 135 | 97-242 | 11 | 107 | 82-164 |
| Calcium (mg/dL) | 9 | 7.9^c^ | 1.7-15.0 | 5 | 7.3 | 2.3-7.9 | 8 | 10.9 | 7.8-11.2 | 11 | 12.4 | 8.1-14.5 |
| Ca:P^a^ | 9 | 0.55 | N/A | 5 | 0.60 | N/A | 8 | 0.86 | N/A | 11 | 0.86 | N/A |
| Cholesterol (mg/dL) | 9 | 396^c^ | 267-596 | 5 | 424 | 267-567 | 8 | 348 | 284-415 | 11 | 367 | 294-525 |
| CK (IU/L)^a^ | 9 | 535 | 14-5,278 | 5 | 272 | 65-1,366 | 8 | 1,022 | 134-4,263 | 11 | 764 | 11-3,601 |
| Glucose (mg/dL) | 9 | 87 | 71-117 | 5 | 92 | 72-98 | 8 | 93 | 79-114 | 11 | 113 | 64-136 |
| LDH (IU/L)^a^ | 9 | 584^c^ | 311-3,640 | 5 | 394 | 338-855 | 8 | 432^d^ | 225-881 | 11 | 234^d^ | 134-431 |
| Phosphorus (mg/dL) | 9 | 14.3 | 10.1-17.9 | 5 | 12.1 | 9.9-15.2 | 8 | 13.3 | 10.5-15.6 | 11 | 12.9 | 10.8-16.4 |
| Potassium (mmol/L) | 9 | 6.3^c^ | 4.2-12.0 | 5 | 6.7 | 6.4-8.8 | 8 | 3.6 | 3.0-4.8 | 11 | 3.5 | 2.9-4.6 |
| Sodium (mmol/L) | 9 | 151^c^ | 146-163 | 5 | 151 | 150-158 | 8 | 147 | 142-151 | 11 | 148 | 146-150 |
| Total protein (g/dL) | 9 | 4.9^c^ | 3.7-5.9 | 5 | 5.3 | 3.8-5.9 | 8 | 3.9 | 3.6-4.4 | 11 | 4.2 | 3.5-5.2 |
| Uric acid (mg/dL) | 9 | 1.0^c^ | 0-2.1 | 5 | 1.8 | 0.7-3.7 | 8 | 0.6 | 0.4-0.9 | 11 | 0.4 | 0.3-1.1 |
| ^a^ ALKP = Alkaline phosphatase, AST = Aspartate aminotransferase, Ca:P = Calcium:phosphorus ratio, CK = Creatine kinase, LDH = Lactate dehydrogenase  ^b^ Harris et al. (2011)  ^c^ Significant difference between foraging females and nesting females  ^d^ Significant difference between nesting females from Costa Rica and nesting females from Papua New Guinea | | | | | | | | | | | | |
